# Automated functional classification of experimental and predicted protein structures

**DOI:** 10.1186/1471-2105-7-278

**Published:** 2006-06-02

**Authors:** Kai Wang, Ram Samudrala

**Affiliations:** 1 Computational Genomics Group, Department of Microbiology, University of Washington, Seattle, WA 98195, USA

## Abstract

**Background:**

Proteins that are similar in sequence or structure may perform different functions in nature. In such cases, function cannot be inferred from sequence or structural similarity.

**Results:**

We analyzed experimental structures belonging to the Structural Classification of Proteins (SCOP) database and showed that about half of them belong to multi-functional fold families for which protein similarity alone is not adequate to assign function. We also analyzed predicted structures from the LiveBench and the PDB-CAFASP experiments and showed that accurate homology-based functional assignments cannot be achieved approximately one third of the time, when the protein is a member of a multi-functional fold family. We then conducted extended performance evaluation and comparisons on both experimental and predicted structures using our Functional Signatures from Structural Alignments (FSSA) algorithm that we previously developed to handle the problem of classifying proteins belonging to multi-functional fold families.

**Conclusion:**

The results indicate that the FSSA algorithm has better accuracy when compared to homology-based approaches for functional classification of both experimental and predicted protein structures, in part due to its use of local, as opposed to global, information for classifying function. The FSSA algorithm has also been implemented as a webserver and is available at .

## Background

It is commonly believed that sequence determines structure, which in turn determines function. This paradigm forms the basis of functional annotation methods using sequence or structure similarity. However, since the structure space is much smaller than either the sequence space or the function space, there will be exceptions to this paradigm: Similar functions may be exerted by distinct sequences and structures, as in the kinase family [1]. Alternately, similar structures may exert very different functions, as in the TIM barrel fold family [2,3]. The presence of multi-functional fold families suggests that structure and function do not always correlate. (Here we refer to "fold family" as a collection of proteins adopting the same structural fold.) However, the presumption among biologists is that the function of protein can be easily inferred whenever its structure is obtained either by experimental means or by computer simulation. This forms part of the rationale for structural genomics projects where the goal is to obtain structures for representative members of a fold family in the hope that the structure and function of the other members of the family will be apparent. While this is true in the majority of the cases, a significant minority (over one third) of structures from structural genomics projects represent proteins of unknown function, annotated merely as "hypothetical proteins" [4]. Classification and identification of the exact function for protein targets given an experimentally determined structure still remains an open challenge [4-6].

For many proteins without experimental structures and easily identifiable sequence homologues, structural models can be generated by fold recognition algorithms and used for functional inference. The fold recognition algorithms typically align a query sequence to proteins whose structures have been experimentally determined, and are extremely effective at determining the correct fold, even when the sequence similarity between the query and its homologue is very low [7,8]. Studies have been conducted to evaluate the possibility of using predicted structures to infer protein function: For example, predicted structures were used to identify possible functional sites through database matching [9,10]. In addition, structure predictions were used to infer function in a genomic scale for proteins without obvious sequence homologues [11-13]. Despite all these studies, the correlation between successful fold recognition and correct functional annotation has not been thoroughly studied and quantitated.

Our first goal was to determine the accuracy of functional inference when the correct structural fold for a given target protein sequence was predicted using fold recognition algorithms. To accomplish this, we evaluated a set of fold predictions made in the LiveBench [14-17] and PDB-CAFASP [18] experiments. We found that similarity in structural folds derived from fold recognition algorithms does not lead to correct functional assignments approximately one third of the time when the protein is a member of a multi-functional fold family. Considering that the structures of most proteins will never be solved experimentally, methods that perform accurate functional annotation based on predicted structure even for this minority of proteins will significantly enhance our ability to utilize the vast amount of available sequence data. Therefore, novel methods to predict function that go beyond sequence and structure comparisons are necessary to reduce the gap between structural genomics and functional genomics.

We previously developed a computational method called Functional Signatures from Structural Alignments (FSSA) [19] to address this problem. In brief, given an ensemble of proteins sharing the same structural fold, we first perform all-against-all structure alignments. We use the alignments to separate the contribution to structure and function for each amino acid residue in each structure using log odds scores. For a given protein, the collection of these log odds scores for all residues comprises its functional signature, which can be used to classify query protein structures into functional categories. Our method shows comparable or better results than other sequence or structure comparison based methods, especially when the sequence identity between a target protein and others belonging to the same fold family is relatively low.

Here, we extend our previous work as follows: We evaluated our algorithm for 42 multi-functional fold families using experimental structures collected from the latest release of the SCOP database (an increase of 28 from the fourteen evaluated previously [19]). We then evaluated the performance of our algorithm using predicted structures generated by the LiveBench and the PDB-CAFASP experiments. In both cases, we showed that our algorithm performs better than sequence and structure comparison approaches for functional annotation. We further investigated the reason for the FSSA algorithm having good performance even on predicted structures that are generated with biases towards the incorrect functional categories (i.e., those that are using a template from a different SCOP superfamily). Finally, we implemented the FSSA algorithm as a webserver [20]. The webserver takes a PDB file and a SCOP fold as input, and outputs predicted SCOP superfamilies and corresponding confidence scores, as well as the functional signature, which indicates the contribution of each position and residue type to the function of the protein.

## Results

### Accuracy of functional assignment based on experimental structure similarity

The Structural Classification of Proteins (SCOP) database curators classify protein domains whose structures or functional features suggest a common evolutionary relationship into the same superfamily [21-23]. We use the SCOP superfamily as a proxy for functional category, since it is generally regarded as a gold standard for defining remote homology and widely used in the literature [13,24,25]. Even though the correlation between SCOP superfamilies and function is not absolute, and that proteins within the same superfamily may have different biochemical activities, this may be used as a reasonable approximation for evaluating functional assignment of classification methods.

We first analyzed the fraction of structural domains that belong to multi-functional fold families, which gives an estimate of how frequently we will encounter the problem of ambiguous functional assignment for a newly solved structure. In the SCOP release version 1.69, 11% of all SCOP folds contain multiple superfamilies, while 46% of all domains belong to one of these multi-functional fold families (Table 1). Therefore, although multi-functional fold families account for a small fraction of the fold space, these folds are usually more abundant than other folds. Our analysis suggests that the problem of ambiguous functional assignment may be encountered for about half of the structures solved experimentally.

### Accuracy of functional assignment based on predicted structure similarity

We next investigated whether functional assignment for a given protein can be inherited from its closest structural homologues predicted by state-of-the-art fold recognition techniques. We collected a set of fold predictions made in the LiveBench [17] and the PDB-CAFASP [18] experiments. These experiments evaluate how well structure prediction servers perform on blind prediction targets. One of the best performing fold recognition methods in these experiments is 3D-Jury [26,27], which collects output from various individual structure prediction servers and generates a consensus prediction. We obtained 86 proteins from the LiveBench 7, LiveBench 8, LiveBench 9 and PDB-CAFASP 1 experiments, representing "hard" prediction targets correctly assigned to a multi-functional fold family.

We then evaluated the correctness of functional assignment for these 86 proteins using their closest structures as determined by 3D-Jury using the SCOP nomenclature (Table 2). The fraction of correct assignments is similar for all four experiments, indicating that our estimates have low variance and high confidence. There is no obvious increase in the fractions of correct assignments for the three consecutive LiveBench experiments, indicating that increasing quality in structure prediction may not necessarily lead to improvements in structure-based annotation transfer. Overall, we found that approximately one-third (26/86 for all four data sets) of the proteins in the multi-functional fold families are not assigned to the correct superfamilies, even when the correct structural folds are identified (Table 2).

### Performance of FSSA on experimental structures

Our published study on FSSA [19] was carried out on a fraction of fold families in the SCOP database (14 fold families where each protein has less than 95% sequence identity to each other). Here we extended our previous performance evaluation to all the 42 SCOP fold families for which sufficient training data are available, and compared the performance of the FSSA algorithm with several other sequence and structure homology based function classification methods (Smith-Waterman, PSI-BLAST, HMM, MAMMOTH and CE). The comparison is not totally equitable since these other methods were not particularly developed or parameterized for functional classification; however, they are widely used by biologists to infer function based on similarity.

**Figure 1:**
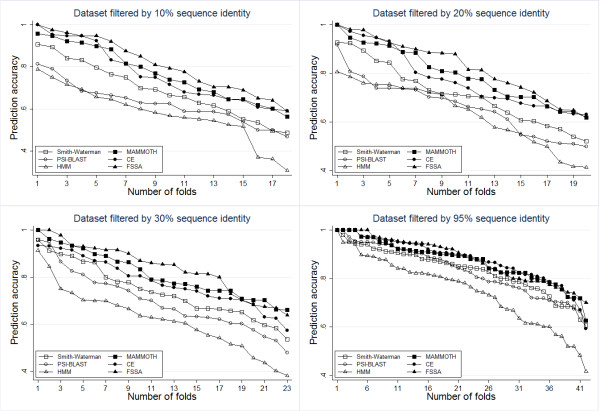
Relative performance of six function classification methods on data sets from the SCOP database that has been filtered by 10%, 20%, 30% and 95% pairwise sequence identity, respectively. We used all folds available (42 fold families in 95% sequence identity level), as opposed to our previous study, where only selected folds in the SCOP database was used (14 fold families in 95% sequence identity level). For each function classification method, the number of SCOP folds is plotted against the minimum prediction accuracy achieved by that method. The FSSA algorithm has the overall best performance in function classification when sequence identity is less than 30%.

To investigate the correlation between performance and similarity among testing and training sequences, we used four different data sets retrieved from the ASTRAL compendium, representing proteins whose pairwise sequence identities are less than 10%, 20%, 30% and 95% to each other. For all sequence identity levels, these structural folds in our data sets contain all-α, all-β, α/β, α+β as well as small proteins, and provide a good representation of the protein fold space. We performed cross-validation experiments to examine the functional classification performance for different methods. Overall, the FSSA algorithm has the best performance when pairwise sequence identity in the data sets is less than 30%, though the differences are subtle between all methods utilizing structural information (Figure 1). Sequence homology based function classification methods perform relatively poorly at low sequence identity levels. Our evaluation demonstrates that the FSSA algorithm would be useful for automated function annotation applications for structural genomics projects, when used in conjunction with other sequence and structure comparison methods.

### Performance of FSSA on predicted structures

**Figure 2:**
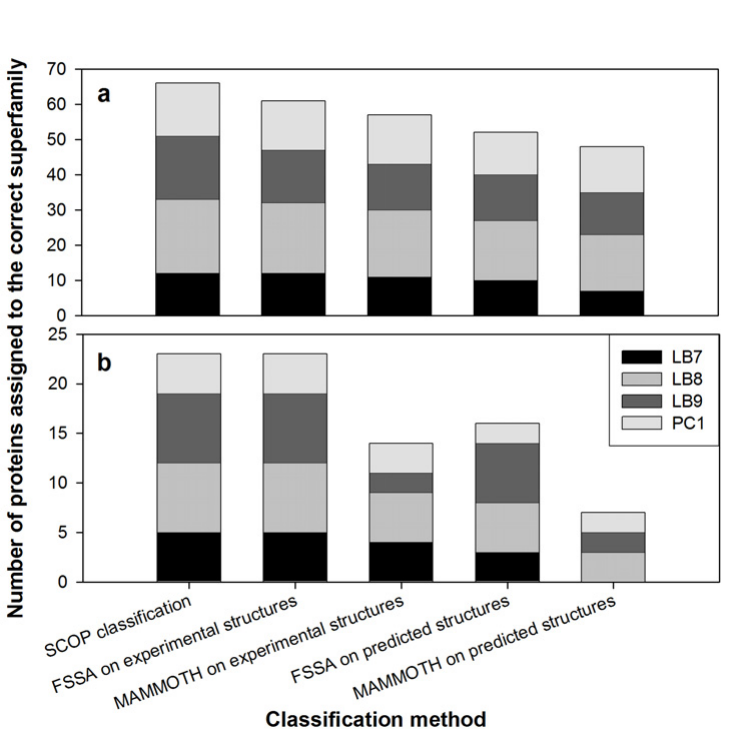
Comparison of function classification performance by FSSA and MAMMOTH on experimental and predicted structures. These structures correspond to selected prediction targets from the LiveBench 7 (LB7), LiveBench 8 (LB8), LiveBench 9 (LB9) and PDB-CAFASP 1 (PC1) experiments. (a) represents those prediction targets that are assigned to the correct SCOP fold (regardless of superfamily) by 3D-Jury; (b) represents those prediction targets that are assigned to the correct SCOP fold but incorrect SCOP superfamily by 3D-Jury. The heights of the first bars ("SCOP classification") in panel (a) and (b) correspond to the total number of targets to be classified for each panel, while the following bars represent the number of targets assigned to correct superfamily by the corresponding prediction methods. The FSSA algorithm has better performance than the structure comparison method for both experimental and predicted structures, and especially for predicted structures that were generated with biases towards the incorrect functional categories.

We next investigated whether the FSSA algorithm can be applied to structures that are predicted by homology modeling techniques. We selected 66 hard prediction targets from the LiveBench and PDB-CAFASP experiments for our analysis. These targets are those that have been assigned to the correct multi-functional fold families by the 3D-Jury system and belong to the 42 fold families for which sufficient training data are available. We used our own homology modeling and optimization algorithms on these prediction targets and generated all-atom structural models (see Methods). We then applied the FSSA algorithm on predicted structures to test whether they can be assigned to the correct functional categories. For comparison, we also tested the experimental structures corresponding to these prediction targets by the FSSA algorithm. We found that both FSSA and structure comparison method perform well, though function predictions on the modeled structures are generally slightly worse than those obtained using the experimental structures (Figure 2 and Additional file 1).

### Performance of FSSA on predicted structures using templates from incorrect SCOP superfamilies

We then focused on 23 structures whose templates (best hits as ranked by the 3D-Jury system) belong to a different superfamily than the query, since these structures are potentially biased towards the incorrect superfamily and pose a challenge for function prediction methods. Structure superposition confirmed that the predicted structures tend to be similar to the templates used to construct the models, with an average C_α _RSMD of 3.42Å. For 16/23 predicted structures, the FSSA algorithm correctly identifies their functional categories, even though the structures were modeled in a manner that biased them towards folds in different function categories. In comparison, the structure comparison method only identifies the correct functional categories for 7/23 predicted structures. This suggests that the FSSA algorithm is less sensitive to biases in predicted structures caused by using templates from different superfamilies.

**Figure 3:**
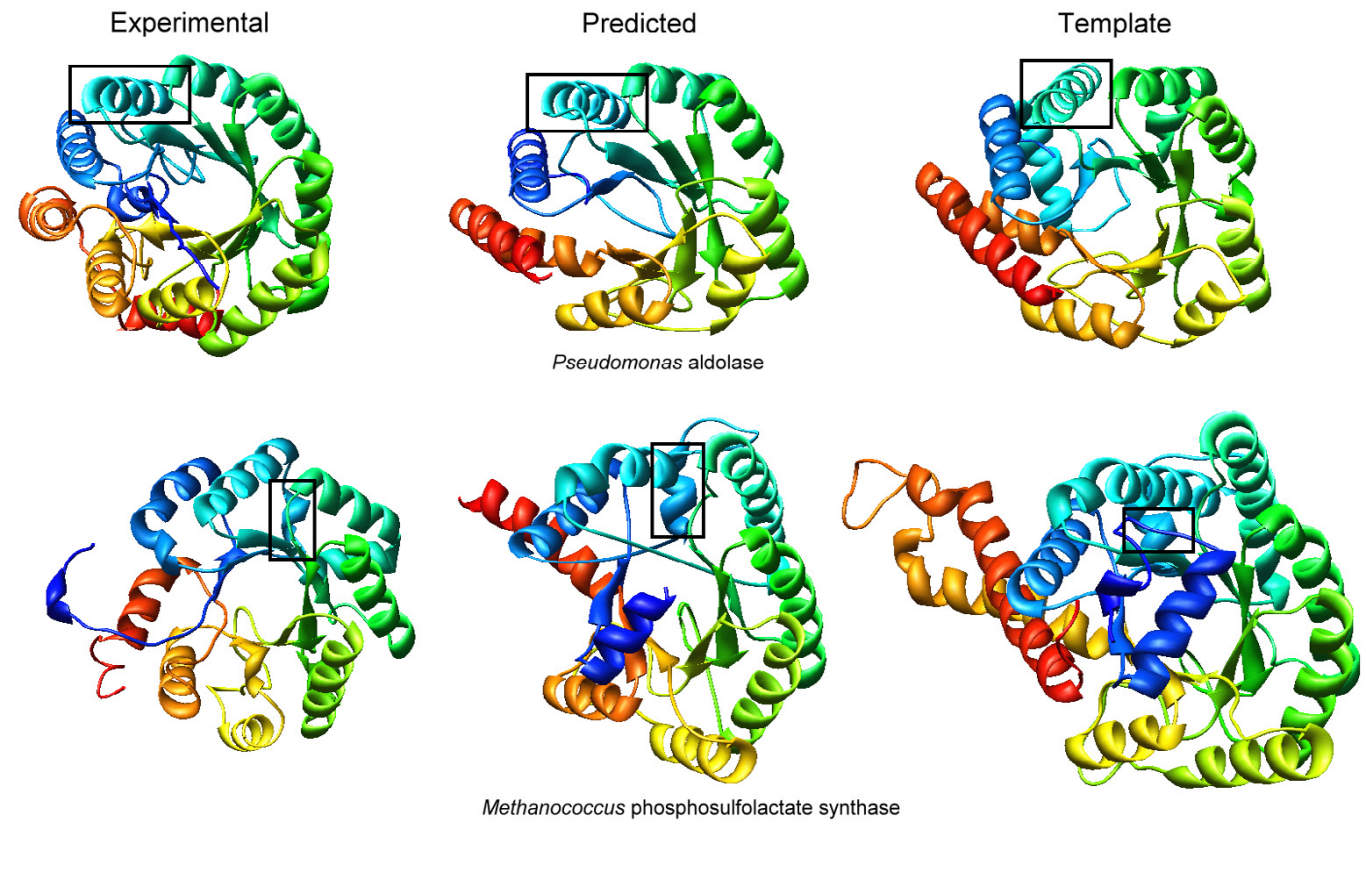
Examples where global protein similarity is not adequate to predict function. Shown are the experimental, predicted, and template structures for protein targets Pseudomonas aldolase (PDB identifier 1nvm-A) and Methanococcus phosphosulfolactate synthase (PDB identifier 1qwg-A) colored by the direction of the chain (blue to red). In both cases, the template and predicted structures have the correctly assigned fold but incorrectly assigned function based on similarity. The predicted structures resemble the template structures overall, but some local features (orientation of the second helix in upper panel and an extra helix-like region in lower panel, shown as black boxes in figure) are more similar to what is observed in the experimental structures. Since the FSSA algorithm uses both local sequence and structure information to determine function, it is less susceptible to such biases in global structure when classifying protein function.

To further investigate the mechanism that enables FSSA to accurately classify modeled structures even when the templates are derived from incorrect SCOP superfamilies, we visually examined two prediction targets: an aldolase from *Pseudomonas *(PDB identifier 1nvm-A) and a phosphosulfolactate synthase from *Methanococcus *(PDB identifier 1qwg-A) (Figure 3). Both targets have 3D-Jury scores higher than 100, indicating high confidence in the accuracy of fold recognition and the alignments generated by the 3D-Jury system. However, the predicted structures for both targets are correctly classified by the FSSA algorithm but not by structure or sequence comparison methods. Both prediction targets belong to the TIM barrel fold, and the predicted structures correctly reproduce the global barrel shape. We found that both predicted structures are generally biased toward the conformation of the template structures, especially in the C-terminal region (shown in red in Figure 3). However, some local structural features in experimental structures are correctly captured by our structure prediction algorithm: For example, the second helix in the predicted structure for the *Pseudomonas *aldolase resembles that of the experimental structure, rather than the template structure. Similarly, for the *Methanococcus *phosphosulfolactate synthase, a small extra helix-like region is correctly generated after the second helix in the barrel, similar to that in the experimental structure. Since the FSSA algorithm uses both local sequence and structure to determine function, it is less susceptible to biases in global structure when classifying protein function.

The good performance of the FSSA algorithm here is mainly due to its immunity to global structural bias, rather than its ability to match functional signatures to the correct superfamily. Our analysis nevertheless suggests that the combination of local structure and local sequence information, rather than global structural fold, is important in assigning function to predicted structures.

### The FSSA algorithm as a webserver

Using data sets from the ASTRAL compendium [28] for the SCOP database, we implemented the FSSA algorithm as a webserver for automated function prediction [20]. Because the FSSA algorithm needs sufficient data for training, currently our server only contains 42 of the 127 multi-functional fold families. Domains in these 42 folds account for 69% of all domains within multi-functional fold families in the SCOP database.

The webserver takes a PDB file and a SCOP fold as input, and outputs predicted SCOP superfamilies and corresponding confidence scores, using the FSSA algorithm as well as sequence and structure comparison methods. It also outputs predicted functional signatures, which indicates the contribution of each position and residue type to the function of the protein.

## Discussion

We have demonstrated and quantitated the degree to which proteins belonging to multi-functional fold families hinder the accurate functional annotation of experimentally derived structures as well as structures modeled by fold recognition methods. Although this situation is relatively well known for structures that have been experimentally solved (for example, those from structural genomics projects [29]), it has not been quantitatively measured for structures modeled by fold recognition methods. In addition, we have also performed extended performance analysis of the FSSA algorithm on both experimental and predicted structures. Our algorithm performs better than structure comparison methods for functional annotation, especially when using modeled structures that are biased towards templates from different functional categories. We further implemented the FSSA algorithm as a webserver so that it is more publicly accessible.

The current implementation of the FSSA algorithm has issues that need to be resolved. The first issue concerns the suitability of using SCOP superfamily to define functional category. Although this manually curated scheme is widely accepted as a proxy for evolutionary relationship, there are many exceptions where proteins with the same superfamily have different functions. Hegyi *et al *has shown that the exact protein function is conserved for 67% of pairs of single domain proteins within the same SCOP superfamily, and for 80% of pairs of multi-domain proteins with the same combination of SCOP superfamilies [30]. Therefore, the SCOP superfamily can be only used to classify broad functional categories or evolutionary relationships, rather than the exact biochemical functions for proteins. The Enzyme Commission (EC) [31] or Gene Ontology (GO) [32] annotations are alternative classification schemes for training our methods. The EC classification can be only applied to enzymes, and for selected structural fold families that contain large numbers of enzymes, such as the TIM barrel fold family, the performance of the FSSA algorithm is similar to what is observed when the SCOP classification scheme is used. Even though some of the structures in the PDB have been assigned computationally identified GO terms through the use of sequence or structural homology [33,34], we cannot use these annotations to train and test our algorithm until a large portion of the PDB contains experimentally verified GO functional assignments. In principle, we could also extend the FSSA algorithm to classify proteins at the family level, rather than superfamily level, allowing for greater specificity in functional annotation. However, since the current SCOP database classifies family level relationships by sequence comparison, it may not be a good reference dataset for training our models. The use of meta-functional signatures from different sources for more detailed and accurate functional classification is being actively explored.

Another issue with the FSSA algorithm concerns our combining multiple small categories into a single "OTHER" category to train our models in a more realistic manner (see Methods). An annotation of "OTHER" however does not shed light on the actual function (except to say that it is not one of the ones already known). In addition, we have noticed that in many cases proteins in the "OTHER" category can be assigned to the correct functional category by the FSSA algorithm, but not by structure comparison methods. In such cases, the good performance of the FSSA algorithm is actually due to its ability to indicate that a given query does not belong to one of the incorrect categories. The problem caused by the "OTHER" category will reduce in severity as the sizes of structural databases increase.

Several structure-based functional annotation systems similar to ours have been developed in recent years [4-6]. For example, protein function can be inferred by scanning a database of 3D templates (set of residues related to function) [35,36]. The Phunctioner method [37] extracts functional sites from multiple structural alignments, and then generates 3D profiles for sets of residues that determine functional specificity. The ProKnow method [38] extracts various sequence, structure and interaction features from structural databases, and relates them to function by annotation profiles. The THEMATICS method [39] identifies enzyme function by computing the theoretical microscopic titration curve for each residue in a protein structure. Our approach markedly differs from these others: (1) The contribution of each amino acid residue to structure and to function is explicitly separated through the analysis of local structure and local sequence. (2) The functional importance of each residue is assigned a quantitative value, rather than a uniform value for selected functionally important residues.

Overall, we envision FSSA as a complementary method to other sequence and structure-based approaches for the annotation of protein function. We believe that the combination and integration of all these methods is necessary to achieve broad annotation of organismal genomes and proteomes.

## Conclusion

Our results indicate that the FSSA algorithm has better accuracy when compared to homology-based approaches for functional classification of both experimental and predicted protein structures, in part due to its use of local, as opposed to global, information for classifying function. Our method can be used in combination with other methods to achieve broad annotation of organismal genomes and proteomes.

## Methods

### Data source

The domain structures and the corresponding sequences for the SCOP database were downloaded from the ASTRAL compendium version 1.69 [28]. Four different sequence subsets were used, representing sequences that have been filtered by 10%, 20%, 30% and 95% pairwise identity by the database curators. A few structures with large missing segments (consecutive C_α _atoms more than 10Å apart) were not used in our study, since structure alignment programs cannot reliably align them.

The prediction targets and their corresponding 3D-Jury predictions [26] for the LiveBench and PDB-CAFASP experiments were downloaded from their corresponding websites at [40] and [41] in July 2005. The primary difference between the two types of experiments is that LiveBench uses proteins with newly deposited structures in the Protein Data Bank (PDB) [42] as targets, while PDB-CAFASP collects pre-released sequences (usually weeks before the experimental structures are released) in the PDB as targets.

### Functional assignments based on predicted structural similarity

To investigate the correlation between successful fold recognition and correct functional assignment, we analyzed hard prediction targets collected from the LiveBench 7, LiveBench 8 and LiveBench 9 and PDB-CAFASP 1 experiments (Table 2). The "hard" prediction targets were defined by the curators of these experiments as those that cannot obtain fold assignments via the PSI-BLAST sequence comparison method. Based on the best hits given by the 3D-Jury system, 86 (from a total of 163) hard targets that belong to multi-functional fold families and have correct fold assignments were used in our study. We analyzed whether these 86 hard targets have correct functional assignments by the 3D-Jury method. An assignment of "correct" for the fold or the function indicates that the target and its closest homologue (as predicted by the 3D-Jury method [26] belong to the same SCOP fold or the same SCOP superfamily, respectively.

### Function classification methods

For each SCOP fold, we combined all superfamilies with less than eight sequences into a single "OTHER" category. We performed four-fold cross-validation experiments on all SCOP folds that contained at least two functional categories. In each of the cross-validation experiments, 75% of the domain structures were used as databases and the remaining 25% structures were used as queries. Each query was assigned to the same functional category as the database sequence with the best "homology score" (E-value for sequence comparison methods, Z-score for structure comparison methods and log odds score for the FSSA algorithm). Sequence-based methods include the Smith-Waterman method with the FASTA package [43], the PSI-BLAST method with the NCBI-BLAST package [44] and the hidden Markov Model methods with the ClustalW program [45] and the HMMER package [46]. For the HMM method, we compiled separate HMM models for each superfamily alignment using hmmbuild with the default parameters and the default hmmls algorithm. We then calibrated these models and used hmmpfam to assign a query sequence to the best scoring model. The structure-based methods include the CE program [47] and the MAMMOTH program [48]. Further details on the function classification experiments are given elsewhere [19].

We strive to solve real-world problems, so we try to make our computational experiments approximate the real-world scenario. There are several marked differences in our evaluation procedures, compared to those used in many publications. First, although the majority of published methods aim at discriminating homologues from structural analogues (binary decision problem), we aim at assigning a given query sequence into a particular functional category (multi-category classification problem), since it reflects the practical problem biologists would face when given a protein of unknown function. Second, unlike others that discard functional categories that contain very few sequences, we combine these small categories into a single "OTHER" category. This makes the correct classifications harder, but it does approximate the real situation in automated function prediction. We believe that the results derived from our evaluation procedures can better approximate the situation for functional annotation of structural genomics targets or modeled structures.

### Structure prediction for targets in LiveBench and PDB-CAFASP experiments

For structure prediction of targets from the LiveBench and PDB-CAFASP experiments, we collected the alignments between the targets and their closest homologues given by the 3D-Jury system. We then used the scgen_mutate program in the RAMP software suite version 0.51 [49] to construct structural models in the following manner: From the alignments generated by the 3D-Jury system, residues that are identical in the target and the template were generated by copying atomic coordinates of the main chain and the side chains, while residues that differ in side chain type (excluding any insertions/deletions) were constructed using a minimum perturbation technique [50,51]. The RAMP software suite was also used for structure preparation, structure superimposition and chain extraction. The molecular visualization was conducted by the UCSF Chimera software [52].

## Availability and requirements

Project name: FSSA server; Project home page:  ; Operating system: platform independent; Programming language: Perl; License: no license required.

## Authors' contributions

KW carried out the computational experiments and drafted the manuscript. RS developed the idea, provided intellectual guidance and mentorship. All authors read and approved the final manuscript.

## Supplementary Material

Additional File 1 Comparative evaluation of three prediction methods (FSSA, MAMMOTH and SSEARCH) on selected prediction targets from the LiveBench 7, LiveBench 8, LiveBench 9 and PDB-CAFASP 1 experiments. This table contains the raw data that is used to generate Figure 2 in the manuscript. Click here for file

## Figures and Tables

**Table 1:** Fraction of multi-functional fold families in the SCOP database. About half of the protein domains belong to a multi-functional fold family, suggesting that the problem of ambiguous functional assignment is very common for experimental structures.

Category	Number of folds	Number of superfamilies	Number of families	Number of domains
Folds with multiple superfamilies	127	755	1,414	32,913
Folds with a single superfamily	1,006	1,006	1,700	37,946

**Table 2:** The fold recognition and functional assignment performance of the 3D-Jury system in the LiveBench 7 (LB7), LiveBench 8 (LB8), LiveBench 9 (LB9) and PDB-CAFASP1 (PC1) experiments. Overall, 43.2% (163/377) of all hard targets in these experiments belong to a multi-functional fold family, similar to the frequency (46.4%) in the SCOP database. Approximately one-third (26/86) of the proteins belonging to a multi-functional fold family are assigned to the incorrect functional category even when the folds are predicted correctly.

**Data Set**	**LB7**	**LB8**	**LB9**	**PC1**	**Total**
Number of targets	115	172	188	130	605
Number of hard targets	73	99	111	94	377
Number of hard targets with correctly identified fold	29	40	36	30	135
Number of hard targets within a multi-functional fold family	33	46	42	42	163
Number of hard targets within a multi-functional fold family with correctly identified fold	15	27	24	20	86
Number of hard targets within a multi-functional fold family with correctly identified function	10	19	16	15	60

## References

[B1] Cheek S, Ginalski K, Zhang H, Grishin NV (2005). A comprehensive update of the sequence and structure classification of kinases. BMC Struct Biol.

[B2] Nagano N, Orengo CA, Thornton JM (2002). One fold with many functions: the evolutionary relationships between TIM barrel families based on their sequences, structures and functions. J Mol Biol.

[B3] Nagano N, Porter CT, Thornton JM (2001). The (betaalpha)(8) glycosidases: sequence and structure analyses suggest distant evolutionary relationships. Protein Eng.

[B4] Watson JD, Laskowski RA, Thornton JM (2005). Predicting protein function from sequence and structural data. Curr Opin Struct Biol.

[B5] Whisstock JC, Lesk AM (2003). Prediction of protein function from protein sequence and structure. Q Rev Biophys.

[B6] Bartlett GJ, Todd AE, Thornton JM, Bourne PE, Weissig H (2003). Inferring protein function from structure. Structural Bioinformatics.

[B7] Godzik A (2003). Fold recognition methods. Methods Biochem Anal.

[B8] Ginalski K, Grishin NV, Godzik A, Rychlewski L (2005). Practical lessons from protein structure prediction. Nucleic Acids Res.

[B9] Zhang B, Rychlewski L, Pawlowski K, Fetrow JS, Skolnick J, Godzik A (1999). From fold predictions to function predictions: automation of functional site conservation analysis for functional genome predictions. Protein Sci.

[B10] Fetrow JS, Skolnick J (1998). Method for prediction of protein function from sequence using the sequence-to-structure-to-function paradigm with application to glutaredoxins/thioredoxins and T1 ribonucleases. J Mol Biol.

[B11] Xu D, Kim D, Dam P, Shah M, Uberbacher E, Xu Y, Setlow JK (2003). Characterization of protein structure and funtion at genome scale using a computational predictiton pipeline. Genetic Engineering: Principles and Methods.

[B12] Pawlowski K, Rychlewski L, Zhang B, Godzik A (2001). Fold predictions for bacterial genomes. J Struct Biol.

[B13] Gough J, Karplus K, Hughey R, Chothia C (2001). Assignment of homology to genome sequences using a library of hidden Markov models that represent all proteins of known structure. J Mol Biol.

[B14] Bujnicki JM, Elofsson A, Fischer D, Rychlewski L (2001). LiveBench-1: continuous benchmarking of protein structure prediction servers. Protein Sci.

[B15] Bujnicki JM, Elofsson A, Fischer D, Rychlewski L (2001). LiveBench-2: large-scale automated evaluation of protein structure prediction servers. Proteins.

[B16] Rychlewski L, Fischer D, Elofsson A (2003). LiveBench-6: large-scale automated evaluation of protein structure prediction servers. Proteins.

[B17] Rychlewski L, Fischer D (2005). LiveBench-8: the large-scale, continuous assessment of automated protein structure prediction. Protein Sci.

[B18] Fischer D, Rychlewski L (2003). The 2002 Olympic Games of protein structure prediction. Protein Eng.

[B19] Wang K, Samudrala R (2005). FSSA: a novel method for identifying functional signatures from structural alignments. Bioinformatics.

[B20] FSSA [http://protinfo.compbio.washington.edu/fssa].

[B21] Murzin AG, Brenner SE, Hubbard T, Chothia C (1995). SCOP: a structural classification of proteins database for the investigation of sequences and structures. J Mol Biol.

[B22] Brenner SE, Chothia C, Hubbard TJ, Murzin AG (1996). Understanding protein structure: using scop for fold interpretation. Methods Enzymol.

[B23] Andreeva A, Howorth D, Brenner SE, Hubbard TJ, Chothia C, Murzin AG (2004). SCOP database in 2004: refinements integrate structure and sequence family data. Nucleic Acids Res.

[B24] Liao L, Noble WS (2003). Combining pairwise sequence similarity and support vector machines for detecting remote protein evolutionary and structural relationships. J Comput Biol.

[B25] Kuang R, Ie E, Wang K, Siddiqi M, Freund Y, Leslie C (2005). Profile-based string kernels for remote homology detection and motif extraction. J Bioinform Comput Biol.

[B26] Ginalski K, Elofsson A, Fischer D, Rychlewski L (2003). 3D-Jury: a simple approach to improve protein structure predictions. Bioinformatics.

[B27] Ginalski K, Rychlewski L (2003). Protein structure prediction of CASP5 comparative modeling and fold recognition targets using consensus alignment approach and 3D assessment. Proteins.

[B28] Chandonia JM, Hon G, Walker NS, Lo Conte L, Koehl P, Levitt M, Brenner SE (2004). The ASTRAL Compendium in 2004. Nucleic Acids Res.

[B29] Burley SK, Almo SC, Bonanno JB, Capel M, Chance MR, Gaasterland T, Lin D, Sali A, Studier FW, Swaminathan S (1999). Structural genomics: beyond the human genome project. Nat Genet.

[B30] Hegyi H, Gerstein M (2001). Annotation transfer for genomics: measuring functional divergence in multi-domain proteins. Genome Res.

[B31] Webb EC (1992). Enzyme Nomenclature 1992.

[B32] Ashburner M, Ball CA, Blake JA, Botstein D, Butler H, Cherry JM, Davis AP, Dolinski K, Dwight SS, Eppig JT, Harris MA, Hill DP, Issel-Tarver L, Kasarskis A, Lewis S, Matese JC, Richardson JE, Ringwald M, Rubin GM, Sherlock G (2000). Gene ontology: tool for the unification of biology. The Gene Ontology Consortium. Nat Genet.

[B33] Ponomarenko JV, Bourne PE, Shindyalov IN (2004). Annotation of 3D Protein Chains in PDB with GO terms via Structural Homology. RECOMB.

[B34] Xie L, Bourne PE (2005). Functional Coverage of the Human Genome by Existing Structures, Structural Genomics Targets, and Homology Models. PLoS Comput Biol.

[B35] Di Gennaro JA, Siew N, Hoffman BT, Zhang L, Skolnick J, Neilson LI, Fetrow JS (2001). Enhanced functional annotation of protein sequences via the use of structural descriptors. J Struct Biol.

[B36] Stark A, Russell RB (2003). Annotation in three dimensions. PINTS: Patterns in Non-homologous Tertiary Structures. Nucleic Acids Res.

[B37] Pazos F, Sternberg MJ (2004). Automated prediction of protein function and detection of functional sites from structure. Proc Natl Acad Sci U S A.

[B38] Pal D, Eisenberg D (2005). Inference of protein function from protein structure. Structure (Camb).

[B39] Ondrechen MJ, Clifton JG, Ringe D (2001). THEMATICS: a simple computational predictor of enzyme function from structure. Proc Natl Acad Sci U S A.

[B40] LiveBench [http://bioinfo.pl/LiveBench].

[B41] PDB-CAFASP [http://bioinfo.pl/Meta/results.pl?B=PDB-Cafasp&V=1].

[B42] Berman HM, Westbrook J, Feng Z, Gilliland G, Bhat TN, Weissig H, Shindyalov IN, Bourne PE (2000). The Protein Data Bank. Nucleic Acids Res.

[B43] Pearson WR (2000). Flexible sequence similarity searching with the FASTA3 program package. Methods Mol Biol.

[B44] Altschul SF, Madden TL, Schaffer AA, Zhang J, Zhang Z, Miller W, Lipman DJ (1997). Gapped BLAST and PSI-BLAST: a new generation of protein database search programs. Nucleic Acids Res.

[B45] Thompson JD, Higgins DG, Gibson TJ (1994). CLUSTAL W: improving the sensitivity of progressive multiple sequence alignment through sequence weighting, position-specific gap penalties and weight matrix choice. Nucleic Acids Res.

[B46] Eddy SR (1998). Profile hidden Markov models. Bioinformatics.

[B47] Shindyalov IN, Bourne PE (1998). Protein structure alignment by incremental combinatorial extension (CE) of the optimal path. Protein Eng.

[B48] Ortiz AR, Strauss CE, Olmea O (2002). MAMMOTH (matching molecular models obtained from theory): an automated method for model comparison. Protein Sci.

[B49] RAMP [http://software.compbio.washington.edu/ramp].

[B50] Hung LH, Samudrala R (2003). PROTINFO: Secondary and tertiary protein structure prediction. Nucleic Acids Res.

[B51] Hung LH, Ngan SC, Liu T, Samudrala R (2005). PROTINFO: New algorithms for enhanced protein structure prediction. Nucleic Acids Res.

[B52] Pettersen EF, Goddard TD, Huang CC, Couch GS, Greenblatt DM, Meng EC, Ferrin TE (2004). UCSF Chimera--a visualization system for exploratory research and analysis. J Comput Chem.

