# Supplementary material for: Automated functional classification of experimental and predicted protein structures
Source: BMC Bioinformatics. 2006 Jun 2;7:278. doi: 10.1186/1471-2105-7-278 (PMC1513613; doi:10.1186/1471-2105-7-278)
Supplement: Additional File 1 — Comparative evaluation of three prediction methods (FSSA, MAMMOTH and SSEARCH) on selected prediction targets from the LiveBench 7, LiveBench 8, LiveBench 9 and PDB-CAFASP 1 experiments. This table contains the raw data that is used to generate Figure 2 in the manuscript. [file 1471-2105-7-278-S1.doc]

Supplementary Table 1. Comparative evaluation of three prediction methods (FSSA, MAMMOTH and SSEARCH) on selected prediction targets from the LiveBench 7 (LB7), LiveBench 8 (LB8), LiveBench 9 (LB9) and PDB-CAFASP 1 (PC1) experiments. The function predictions were made by the FSSA server. The numbers in the cell indicate the fraction of targets that were assigned to the correct functional category. The FSSA algorithm has better performance than the structure comparison method on both experimental and predicted structures, especially for predicted structures that were generated using a template belonging to the incorrect superfamily.

| **METHOD** | **Structure** | **LB7** | **LB8** | **LB9** | **PC1** | **Total** |
| --- | --- | --- | --- | --- | --- | --- |
| **Targets that are assigned to the correct SCOP folds by 3D-Jury** | | | | | | |
| FSSA | Predicted | 10/12 | 17/21 | 13/18 | 12/15 | 52/66 |
| MAMMOTH | Predicted | 7/12 | 16/21 | 12/18 | 13/15 | 48/66 |
| FSSA | Experimental | 12/12 | 20/21 | 15/18 | 14/15 | 61/66 |
| MAMMOTH | Experimental | 11/12 | 19/21 | 13/18 | 14/15 | 57/66 |
| SSEARCH |  | 10/12 | 18/21 | 13/18 | 12/15 | 53/66 |
| **Targets that are assigned to the correct SCOP folds but incorrect superfamilies by 3D-Jury** | | | | | | |
| FSSA | Predicted | 3/5 | 5/7 | 6/7 | 2/4 | 16/23 |
| MAMMOTH | Predicted | 0/5 | 3/7 | 2/7 | 2/4 | 7/23 |
| FSSA | Experimental | 5/5 | 7/7 | 7/7 | 4/4 | 23/23 |
| MAMMOTH | Experimental | 4/5 | 5/7 | 2/7 | 3/4 | 14/23 |
| SSEARCH |  | 3/5 | 4/7 | 4/7 | 1/4 | 12/23 |
